# 602. Intravenous Push Versus Intravenous Piggyback Administration of Cephalosporin Antibiotics: Impact on Safety, Workflow, and Cost

**DOI:** 10.1093/ofid/ofab466.800

**Published:** 2021-12-04

**Authors:** Ryan Lee, Thuong Tran, Susanna Tan, Patricia Chun

**Affiliations:** 1 VA Long Beach, Rancho Palos Verdes, CA; 2 Veterans Affairs Long Beach Medical Center, Long Beach, CA; 3 VA Long Beach Healthcare System, Long Beach, CA

## Abstract

**Background:**

IV piggyback (IVPB) infusion has been the standard method of administration of IV antibiotics since the 1970s. Literature has demonstrated that the IV push (IVP) method has similar pharmacokinetic exposures and risk for complications as short infusion IVPB, and may have potential benefits. The primary objective is to evaluate the incidence of infusion-related complications in patients receiving cefazolin, ceftriaxone, and cefepime administered via IVP versus short infusion IVPB at the Veteran Affairs Long Beach Healthcare System. The secondary objectives include evaluating the time-to-onset of complications, time-to-first-dose combination vancomycin in the emergency department (ED), cost, and nursing and pharmacy staff preference between IVP and IVPB.

**Methods:**

This is a retrospective, single-center cohort study. Patients who received ceftriaxone, cefepime, or cefazolin between April 1^st^, 2019 – December 31^st^, 2019, and April 1^st^, 2020 – December 31^st^, 2020 were included. Patients who received the study antibiotics via IVPB during the IVP period were excluded. Statistical analyses were performed using the chi-square, fisher’s exact, Mann-Whitney U, and unpaired t-tests where appropriate. Complications associated with IVP or IVPB administration were assessed via chart review of electronic health records. Surveys to nursing and pharmacy staff were distributed using Microsoft Forms.

**Results:**

366 treatment episodes were evaluated for 355 unique patients. Complications occurred in 13 out of 183 (7.1%) treatment episodes in the IVP group compared to 18 out of 183 (9.8%) treatment episodes in the IVPB group (P = 0.35). The median time to complications was 2 days for both groups. IVP cefepime and ceftriaxone reduced the median time-to-first-dose vancomycin in the ED by 25 minutes. The use of cefazolin, ceftriaxone, and cefepime as IVP yielded a quarterly cost savings of &38,890.04. 55% of nursing staff and 85% of pharmacy staff prefer IVP administration for cefazolin, ceftriaxone, and cefepime.

**Conclusion:**

Cefazolin, ceftriaxone, and cefepime given as IVP were observed to be as safe as IVPB while reducing time-to-first dose vancomycin in the ED and cost, and is the preferred method of administration among nursing and pharmacy staff.

**Disclosures:**

**All Authors**: No reported disclosures

